# Clinical utility of 12‐lead electrocardiogram in evaluating heart disease in patients with muscular dystrophy: Assessment of left ventricular hypertrophy, conduction disease, and cardiomyopathy

**DOI:** 10.1111/anec.12876

**Published:** 2021-07-11

**Authors:** Anish Nikhanj, Haran Yogasundaram, Shane Kimber, Zaeem A. Siddiqi, Gavin Y. Oudit

**Affiliations:** ^1^ Division of Cardiology; ^2^ Mazankowski Alberta Heart Institute; ^3^ Division of Neurology Department of Medicine University of Alberta Edmonton Canada

**Keywords:** electrocardiogram, left bundle branch block, left ventricular hypertrophy, muscular dystrophy, QRS fragmentation

## Abstract

**Introduction:**

Heart disease remains a leading cause of mortality in patients with muscular dystrophy (MD), and cardiac assessment by standard imaging modalities is challenging due to the prominence of physical limitations.

**Methods:**

In this prospective cohort study of 169 MD patients and 34 negative control patients, we demonstrate the clinical utility of a 12‐lead electrocardiogram (ECG) as an effective modality for the assessment of cardiac status in patients with MD. We assessed the utility of conventional criteria for electrocardiogram‐indicated left ventricular hypertrophy (ECG‐LVH) as well as ECG morphologies.

**Results:**

Cornell voltage, Cornell voltage‐duration, Sokolow–Lyon voltage, and Romhilt‐Estes point score criteria demonstrated low sensitivity and minimal positive predictive value for ECG‐LVH when compared with cardiac imaging. Patients with LBBB had a high probability of a cardiomyopathy (relative risk [RR], 2.75; 95% confidence interval [CI], 2.14–3.53; *p* < .001), and patients with QRS fragmentation (fQRS) had a high probability of a cardiomyopathy (RR, 1.76; 95% CI, 1.20–2.59; *p* = .004), requiring cardiac medication and device intervention. We found that an R/S ratio >1 in V1 and V2 is highly specific (specificity, 0.89; negative predictive value [NPV], 0.89 and specificity, 0.82; NPV, 0.89, respectively) for patients with dystrophinopathies compared with other types of MD.

**Conclusion:**

The identification of LBBB and fQRS was linked to cardiomyopathy in patients with MD, while ECG‐LVH was of limited utility. Importantly, these findings can be applied to effectively screen a broad cohort of MD patients for structural heart disease and prompt further evaluation and therapeutic intervention.

## 1 INTRODUCTION

Heart disease remains a leading cause of mortality in patients with muscular dystrophy (MD). (Mascarenhas et al., 1994; Mathieu et al., 1999; Nikhanj et al., 2020; Verhaert et al., 2011; Wexberg et al., 2016) Patient condition and management are often complicated by respiratory, neurological, and metabolic comorbidities, and clinical assessment is challenged by progressive muscle weakening and wasting, obesity, wheelchair dependence, and respiratory aids. Patients with dystrophinopathies including Duchenne muscular dystrophy (DMD) and Becker's muscular dystrophy (BMD), limb‐girdle muscular dystrophy (LGMD), type 1 myotonic dystrophy (DM1), and facioscapulohumeral muscular dystrophy (FSHD) are distinguished by their burden of heart disease. (Benhayon et al., 2015; Miskew Nichols et al., 2018; Nikhanj et al., 2019; Nikhanj, Yogasundaram, et al., 2020) 12‐lead electrocardiogram (ECG) is an accessible and practical modality for cardiac assessment. Morphology such as electrocardiogram‐indicated left ventricular hypertrophy (ECG‐LVH) defined by specific criteria has been shown to be associated with heart disease, heart failure, and adverse clinical outcomes including mortality. (Aro Aapo & Chugh, 2016; Kannel et al., 1969; Levy et al., 1990) Left bundle branch block (LBBB) is a predictor of mortality and is linked to LV systolic dysfunction. (Baldasseroni et al., 2002; Iuliano et al., 2002) Additionally, QRS fragmentation (fQRS) has been associated with major adverse cardiac events (MACE) including life‐threatening arrhythmias and mortality in patients with heart disease, (Das et al., 2008; Terho et al., 2014) as well as an association with systolic dysfunction in patients with DMD. (Cho et al., 2017; Yoo et al., 2017) We identified ECG‐LVH, LBBB, and fQRS as common and recurrent ECG features in our heterogenous MD patient cohort, and we investigated their clinical utility for the front‐line cardiac assessment of patients with MD.

## 2 METHODS

### 2.1 Study population

One hundred and sixty‐nine patients with MD were recruited from the Neuromuscular Multidisciplinary (NMMD) clinic at the Kaye Edmonton Clinic, University of Alberta (Edmonton, Canada). All patients received a baseline 12‐lead ECG study with a subsequent transthoracic echocardiogram (TTE) or cardiac magnetic resonance (CMR) imaging study within 6 months. Thirty‐four age‐ and gender‐matched patients with non‐MD myopathies were recruited to serve as a negative control cohort for heart disease, as previously described. (Nikhanj, Yogasundaram, et al., 2020) Patients were prospectively tracked over a median follow‐up period of 1.88 (interquartile range [IQR], 1.21–2.25) years between November 5, 2014, and November 9, 2020. Our cohort included patients with a dystrophinopathy (26 DMD and 10 BMD patients), LGMD (36 patients), DM1 (74 patients), and FSHD (23 patients), as confirmed by genetic testing. All clinical data including the use of medical therapy and device intervention were obtained by electronic chart review. Patients were referred to the NMMD clinic and recruited to our study at various stages of their disease, and all patients provided informed and written consent at study enrollment. The investigation was approved by the Health Research Ethics Board at the University of Alberta.

### 2.2 12‐lead electrocardiogram

All patients were assessed using a Philips PageWriter TC70 Cardiograph (Philips Healthcare, Amsterdam, the Netherlands) ECG system as part of routine clinical care. Patients with mild and moderate ambulatory status were assessed in a supine position, while wheelchair‐bound patients remained sitting. All ECGs were interpreted by the attending cardiologist as part of patient clinical care and followed by a blinded analysis of ECG morphology and intervals using Cardio Calipers version 3.3 digital caliper software (Iconico Inc.,). Standard interval measurements were captured and corrected QT intervals were acquired using Bazett (Bazett, 1920), Fridericia (Fridericia, 2003), Framingham (Sagie et al., 1992), and Hodges (Hodges et al., 1983) formulae. We also applied the recently proposed corrective QT interval formula by Tang & Rabkin to patients with LBBB. (Tang & Rabkin, 2019) On the basis of unique R‐wave progression documented in patients with dystrophinopathies, namely DMD, (Sanyal et al., 1978) we assessed the performance of R‐wave patterns such as R‐wave amplitude in V1 > V2, R/S in V1 >1.00 and >1.50, and R/S in V2 > 1.00 and 1.50, for their ability to differentiate patients diagnosed with a dystrophinopathy from the broader cohort. Electrocardiogram axis and segmental variants such as J‐point elevation were noted and atrioventricular block (AVB), left anterior fascicular block (LAFB), left posterior fascicular block (LPFB), LBBB, right bundle branch block (RBBB), and nonspecific intraventicular conduction delay (IVCD) was also captured. (Kusumoto et al., 2019; Yancy Clyde et al., 2017) All incidences of atrial and ventricular tachyarrhythmias were documented.

We defined ECG‐LVH using the Cornell voltage criteria (CV), Cornell voltage‐duration product criteria (CP), Sokolow–Lyon voltage criteria (SL), and the Romhilt‐Estes point score system (RE), which are conventional criteria used in clinical practice (Table S1). We defined LBBB as a QRS duration >120 ms, accompanied by an absence of Q waves in the lateral leads, slurred R waves in leads I and aVL, and RSR’ pattern in V5 and V6 with R peak time greater than 60 ms. (Kusumoto et al., 2019) We defined fQRS as an RSR’ pattern in 2 contiguous anterior, lateral, or inferior leads; or notches in the nadir of R or S waves in 2 contiguous leads. (Cho et al., 2017) For patients with bundle branch block, fQRS was defined as RSR’ patterns in more than 2 contiguous leads or notches in the nadir of R or S waves in more than 2 contiguous leads. (Das Mithilesh et al., 2008) Electrocardiogram‐indicated left ventricular hypertrophy was compared with anatomical measures of left ventricular (LV) mass obtained from subsequent TTE or CMR, from which LA volume was also obtained, and both were evaluated relative to guideline‐defined reference ranges. (Kawel‐Boehm et al., 2015; Lang et al., 2015) Cardiomyopathy was defined as left ventricular ejection fraction (LVEF) <55% or a left ventricular end‐diastolic volume index (LVEDVi) >105 mL/m², (Kawel‐Boehm et al., 2015) and LVEF could be obtained from either TTE or CMR given the previously demonstrated concordance. (Nikhanj, Yogasundaram, et al., 2020).

Serial tracking of ECG parameters occurred from baseline (*n* = 203), follow‐up 1 (*n* = 153; median follow‐up of 1.08 [IQR, 0.86–1.51] years from baseline), and follow‐up 2 (*n* = 89; median follow‐up of 1.02 [IQR, 0.85–1.22] years from follow‐up 1). The trailing number of patients at each follow‐up period reflected ongoing patient enrollment and the prospective nature of the study. Serial data tracking facilitated the analysis of ECG parameter changes over time among MD patients with cardiomyopathy, MD patients without cardiomyopathy, and patients with non‐MD myopathies.

### 2.3 Statistical methods

Continuous variables were compared using a Mann‐Whitney U test or Kruskal–Wallis test, and all categorical data were compared using Pearson's chi‐square tests. Criteria used to qualify ECG‐LVH were assessed relative to corresponding cardiac imaging studies and compared using performance metrics such as sensitivity and specificity to evaluate criteria accuracy, as well as positive predictive value (PPV) and negative predictive value (NPV) in consideration of the prevalence of left ventricular hypertrophy (LVH). A relative risk (RR) assessment with a 95% confidence interval (CI) was used to compare the probabilities of cardiac outcomes between groups defined by the presence or absence of LBBB and fQRS. Multivariate fixed effect models (adjusted for age, gender, cardiac medication use, and cardiac device intervention, with consideration for variable follow‐up periods) were used to compare serial changes in ECG parameters among defined groups. All statistical analyses were performed in R version 4.0.3, and a *p*‐value <0.05 was considered significant.

## 3 RESULTS

### 3.1 Clinical characteristics of cohorts

The median age of our composite MD cohort was 36 (interquartile range [IQR], 23.5–49.5) years, which included 64 (37.9%) females (Figure S1 (a)). The dystrophinopathies cohort was comprised of patients that were exclusively male and notably young (Table 1). These patients exhibited profound skeletal muscle weakness and wasting as well as a high prevalence of respiratory disease and sleep‐disordered breathing (SDOB) (Table 1). The LGMD and DM1 cohorts were evenly comprised of males and females with a high prevalence of comorbidities such as diabetes, dyslipidemia, hypertension, and respiratory disease, as seen in patients with FSHD (Table 1). Our non‐MD myopathies cohort had a median age of 47 (IQR, 29.0–60.0), which included 16 (47.1%) females, with a notable prevalence of comorbidities comparable to those documented in the MD cohorts (Table 1). Similarly, respiratory disease was diagnosed in 18 (52.9%) patients and SDOB was diagnosed in 7 (20.6%) patients.

**TABLE 1:** Baseline characteristics

Characteristic	Dystrophinopathies (*n* = 36)	LGMD (*n* = 36)	DM1 (*n* = 74)	FSHD (*n* = 23)	Non‐MD Myopathies (*n* = 34)	*p*‐value^a^	*p*‐value^b^
Males/Females, No.	36 (100.0)	18 (50.0)/18 (50.0)	37 (50.0)/37 (50.0)	14 (60.9)/9 (39.1)	18 (52.9)/16 (47.1)	<.001	.32
Median Age, Yrs	22.0 (18.0–28.8)	39.0 (23.0–56.3)	42.0 (33.0–50.0)	45.0 (25.8–53.5)	47.0 (29.0–60.0)	<.001	.08
Current/Former Smoker, No.	1 (2.78)	5 (13.9)	16 (21.6)	3 (13.0)	4 (11.8)	.12	.65
Comorbidities, No.							
Diabetes	0	8 (22.2)	6 (8.11)	3 (13.0)	5 (14.7)	.03	.43
Dyslipidemia	1 (2.78)	4 (11.1)	10 (13.5)	4 (17.4)	8 (23.5)	.13	.05
Hypertension	2 (5.56)	7 (19.4)	4 (5.41)	6 (26.1)	7 (20.6)	.016	.14
Respiratory Disease	26 (72.2)	7 (19.4)	40 (54.1)	6 (26.1)	18 (52.9)	<.001	.51
SDOB	14 (38.9)	4 (11.1)	22 (29.7)	7 (30.4)	7 (20.6)	.08	.38
Anemia	3 (8.33)	2 (5.56)	0	0	0	.04	‐‐
Vitals, median							
HR, bpm	82.0 (75.0–100.0)	72.0 (70.0–82.0)	70.0 (64.0–80.0)	78.0 (66.5–80.0)	75.0 (70.0–80.0)	.021	.84
sBP, mmHg	107.5 (100.8–121.8)	125.0 (114.0–137.0)	114.0 (106.0–122.5)	132.0 (121.0–139.5)	126.0 (120.0–137.0)	<.001	.002
dBP, mmHg	71.0 (64.3–78.0)	80.0 (71.0–87.0)	74.0 (67.5–78.0)	84.0 (77.0–88.0)	76.0 (72.0–84.0)	<.001	.18
Serum Chemistry, median							
BNP, pg/mL	20.0 (9.50–68.0)	24.0 (11.0–45.0)	22.0 (14.0–35.0)	16.5 (10.0–22.5)	19.0 (13.0–27.0)	.71	.98
CK, U/L	1277.0 (377.8–2380.8)	708.5 (322.0–2353.8)	237.0 (144.8–308.3)	174.5 (146.0–452.3)	195.0 (45.8–408.3)	<.001	.005
Creatinine, µmol/L	20.0 (15.8–31.0)	41.0 (29.0–59.0)	57.0 (50.8–69.5)	48.0 (39.0–70.0)	55.5 (29.5–66.0)	<.001	.73
Potassium, mmol/L	4.30 (4.05–4.50)	4.10 (3.80–4.35)	4.30 (3.90–4.50)	4.30 (4.00–4.60)	4.10 (3.90–4.30)	.57	.23

Note

Values are presented as median (interquartile range) or n (%). ^a^Indicates statistical analysis comparing all patients. ^b^Indicates statistical analysis comparing the cohorts of patients with muscular dystrophy (MD) and non‐MD myopathies. Abbreviations: BNP, B‐Type natriuretic peptide; CK, creatine kinase; dBP, diastolic blood pressure; DM1, type 1 myotonic dystrophy; FSHD, facioscapulohumeral muscular dystrophy; HR, heart rate; LGMD, limb‐girdle muscular dystrophy; sBP, systolic blood pressure; SDOB, sleep‐disordered breathing.

### 3.2 Burden of cardiomyopathy and arrhythmias

The broader MD cohort had a high prevalence of cardiomyopathy, which was diagnosed in 68 (40.2%) of the MD patients (Figure S1 (b)). Patients with dystrophinopathies showed LV and right ventricular (RV) dilation, elevated LV mass, and a marked reduction in biventricular systolic function (Table 2), and 28 (77.8%) patients were diagnosed with cardiomyopathy. Twelve of 22 (54.5%) patients that received CMR had evidence of myocardial fibrosis. Patients with LGMD exhibited a comparable prevalence of structural heart disease as well as a reduction in LVEF (Table 2), and 13 (36.1%) patients were diagnosed with a cardiomyopathy. Seven of 22 (31.8%) patients that received CMR had evidence of myocardial fibrosis. Patients with DM1 exhibited normal cardiac structure and reduced median LVEF (Table 2), and cardiomyopathy was diagnosed in 22 (29.7%) patients. One of 22 (4.55%) patients that received CMR had evidence of myocardial fibrosis. Patients with FSHD showed normal LV size and biventricular systolic function, though RV diastolic volumes were elevated (Table 2), and accordingly 5 (14.7%) patients were diagnosed with cardiomyopathy. One of 14 (7.14%) patients that received CMR had evidence of myocardial fibrosis.

**TABLE 2:** Baseline cardiac assessment

Modality	Dystrophinopathies	LGMD	DM1	FSHD	Non‐MD Myopathies	*p*‐value^a^	*p*‐value^b^
12‐Lead ECG	(*n *= 36)	(*n *= 36)	(*n* = 74)	(*n* = 23)	(*n* = 34)		
Heart Rate, bpm	79.5 (71.0–97.3)	70.5 (60.0–82.0)	69.0 (62.0–80.0)	70.0 (59.5–83.5)	74.0 (67.3–80.8)	.025	.68
RR Interval, ms	790.0 (647.0–895.0)	910.5 (750.0–1025.5)	889.0 (763.0–1024.0)	856.0 (716.0–1004.0)	826.5 (734.3–896.0)	.07	.32
PR Interval, ms	132.0 (120.0–137.0)	148.0 (133.0–160.0)	190.0 (172.0–221.0)	164.0 (144.5–172.5)	155.0 (140.0–180.0)	<.001	.73
QRS Duration, ms	95.5 (89.5–109.3)	97.0 (90.5–109.5)	107.5 (96.3–121.5)	92.0 (86.0–96.0)	95.5 (86.0–108.0)	<.001	.18
QT Interval, ms	378.5 (360.5–396.0)	394.0 (372.5–426.0)	411.5 (390.3–436.0)	378.0 (357.5–407.0)	388.0 (380.0–419.0)	<.001	.80
Corrected QT Interval (Bazett), ms	430.9 (410.9–451.4)	426.6 (401.5–451.6)	438.0 (417.1–453.0)	424.0 (408.5–433.3)	437.7 (419.8–453.2)	.17	.40
Corrected QT Interval (Framingham), ms	378.5 (360.5–395.9)	394.0 (372.5–426.0)	411.5 (390.3–436.0)	378.0 (357.6–407.0)	388.0 (380.0–419.0)	<.001	.79
Corrected QT Interval (Fridericia), ms	412.1 (388.9–428.2)	409.6 (399.6–437.8)	429.4 (410.6–442.0)	409.6 (395.2–422.6)	424.4 (405.7–435.8)	.007	.54
Corrected QT Interval (Hodges), ms	415.0 (401.7–441.7)	415.9 (402.9–434.6)	429.1 (414.3–444.7)	407.8 (393.9–421.9)	420.9 (402.9–436.7)	.001	.88
QRS Axis	64.0 (11.3–100.3)	46.0 (18.0–82.8)	13.0 (−28.0–50.0)	48.0 (16.0–67.5)	32.0 (−4.75–65.3)	<.001	.84
T‐wave Axis	47.0 (15.0–75.0)	47.5 (29.3–70.5)	51.0 (37.0–61.3)	49.0 (26.8–63.8)	34.0 (25.5–50.8)	.12	.012
QRS Fragmentation	6 (16.7)	2 (5.56)	11 (23.4)	1 (4.35)	0	.06	‐‐
J‐Point Elevation	6 (16.7)	6 (16.7)	7 (9.46)	2 (8.70)	3 (8.82)	.64	.55
1° AVB	0	2 (5.56)	19 (25.7)	0	2 (5.88)	<.001	.27
LAFB	2 (5.56)	0	7 (9.46)	2 (8.70)	1 (2.94)	.31	.42
LPFB	0	0	0	1 (4.35)	0	‐‐	‐‐
LBBB	2 (5.56)	0	15 (20.3)	0	0	<.001	‐‐
RBBB	1 (2.78)	0	1 (1.35)	0	1 (2.94)	.77	.44
IVCD	4 (11.1)	3 (8.33)	7 (9.46)	2 (8.70)	3 (8.82)	.99	.91
							
Echocardiogram	(*n* = 31)	(*n *= 29)	(*n* = 71)	(*n* = 20)	(*n* = 28)		
LA Vol Index, mL/m^2^	22.4 (15.5–34.1)	20.8 (14.7–25.1)	18.6 (14.9–23.9)	19.1 (14.4–21.6)	17.6 (15.1–27.9)	.78	.78
LVIDd, cm	4.64 (4.14–5.41)	4.77 (4.41–5.20)	4.40 (4.06–4.85)	4.40 (4.00–4.80)	4.52 (3.80–5.00)	.06	.96
LVIDs, cm	3.49 (2.87–4.42)	3.36 (2.84–3.95)	2.80 (2.60–3.10)	2.83 (2.50–3.05)	2.80 (2.61–3.30)	<.001	.28
LVPWd, cm	0.72 (0.66–0.76)	0.87 (0.73–0.98)	0.81 (0.73–1.00)	0.80 (0.73–0.88)	0.82 (0.77–0.97)	.014	.32
LVEF, %	40.0 (25.0–53.9)	55.0 (54.4–60.0)	55.0 (55.0–60.0)	60.0 (55.0–60.0)	55.0 (55.0–60.0)	<.001	.71
LVMI, g/m^2^	80.4 (63.0–93.3)	74.9 (63.3–84.0)	65.3 (54.9–80.5)	67.3 (57.5–76.2)	67.4 (58.7–77.3)	.11	.64
Cardiac MRI	(*n* = 18)	(*n* = 22)	(*n* = 22)	(*n* = 13)	(*n* = 14)		
LA Vol Index, mL/m^2^	38.0 (33.0–43.0)	33.4 (28.2–38.2)	29.0 (24.8–33.2)	31.6 (28.0–34.4)	39.8 (31.2–50.4)	.13	.06
LVEDVi, mL/m^2^	94.0 (82.3–110.8)	77.5 (65.3–96.8)	62.5 (55.5–72.8)	74.0 (56.0–85.0)	77.0 (64.5–83.3)	<.001	.88
LVESVI, mL/m^2^	50.0 (36.0–63.0)	35.0 (26.0–46.0)	29.0 (21.0–37.0)	26.5 (21.3–43.5)	27.0 (22.5–37.3)	.005	.15
LVEF, %	45.0 (39.3–56.5)	55.0 (47.5–58.0)	56.5 (50.3–62.8)	58.0 (53.0–68.0)	61.5 (57.0–65.0)	.004	.012
LVMI, g/m^2^	59.0 (45.0–67.0)	48.0 (40.0–60.0)	42.0 (38.0–49.0)	42.0 (37.0–50.0)	52.5 (44.3–61.5)	.035	.27
RVEDVi, mL/m^2^	78.0 (68.0–93.0)	68.0 (62.0–79.0)	61.0 (56.5–75.0)	79.5 (57.3–92.5)	74.5 (69.0–80.0)	.10	.53
RVESVi, mL/m^2^	38.5 (35.3–47.8)	33.0 (28.0–42.0)	31.0 (27.0–35.0)	39.0 (26.0–45.0)	34.0 (29.5–39.3)	.19	.69
RVEF, %	49.0 (44.0–54.0)	52.0 (49.0–54.0)	51.0 (47.0–55.0)	53.0 (51.3–55.5)	56.5 (49.0–58.5)	.14	.031

Note

Values are presented as median (interquartile range) or n (%). ^a^Indicates statistical analysis comparing all patients. ^b^Indicates statistical analysis comparing the cohorts of patients with muscular dystrophy (MD) and non‐MD myopathies. Abbreviations: 1° AVB, first‐degree atrioventricular block; DM1, type 1 myotonic dystrophy; FSHD, facioscapulohumeral muscular dystrophy; IVCD, intraventricular conduction delay; LA Vol Index, left atrial volume index; LAFB, left anterior fascicular block; LBBB, left bundle branch block; LGMD, limb‐girdle muscular dystrophy; LPFB, left posterior fascicular block; LVEDVi, left ventricular end‐diastolic volume index; LVEF, left ventricular ejection fraction; LVESVi, left ventricular end‐systolic volume index; LVIDd, left ventricular internal dimension at end‐diastole; LVIDs, left ventricular internal dimension at end‐systole; LVMI, left ventricular mass index; LVPWd, left ventricular posterior wall thickness at end‐diastole; RBBB, right bundle branch block; RVEDVi, right ventricular end‐diastolic volume index; RVEF, right ventricular ejection fraction; RVESVi, right ventricular end‐systolic volume index.

Arrhythmias were documented in patients with DMD including atrial flutter in 1 patient and ventricular tachycardia (VT) in 5 patients. Four patients with LGMD had atrial fibrillation or flutter, and VT was reported in 2 patients. Patients with DM1 had a high incidence of arrhythmias as atrial fibrillation or flutter was reported in 16 patients and VT reported in 8 patients. There were no arrhythmias reported in patients with FSHD. Seven patients from our MD cohort received an implantable cardiac defibrillator, and 19 patients received pacemaker therapies, including 13 patients receiving cardiac resynchronization therapy (CRT) devices for LBBB.

### 3.3 Differences in 12‐lead electrocardiogram features

Patients with dystrophinopathies showed parameters within normal limits though PR intervals were relatively shortened at 132.0 (IQR, 120.0–137.0) ms (Table 2). J‐point elevation was a common finding and there was a notable prevalence of fQRS in 6 (16.7%) patients (Figure S2) and ventricular conduction delays including LBBB in 2 (5.56%) patients (Table 2, Figure S3). Patients with LGMD exhibited similar ECG findings, though there was a markedly low prevalence of conduction delays in these patients (Table 2). Patients with FSHD had parameters within normal limits with a low prevalence of conduction delays (Table 2).

Patients with DM1 were distinguished by their abnormal ECG studies, which showed prolonged PR intervals at 190.0 (IQR, 172.0–221.0) ms, QRS duration at 107.5 (IQR, 96.3–121.5) ms, and a QRS axis at 13.0 (IQR, −28.0–50.0) degrees (Table 2). Standard QT intervals and corrected QT intervals, not including the Bazett formula corrected QT interval, were prolonged (Table 2). QRS fragmentation was visualized in 11 (23.4%) patients (Figure S4), and conduction delays were prevalent as first‐degree AVB in 19 (25.7%) patients, LAFB in 7 (9.46%) patients, LBBB in 15 (20.3%) patients (Figure S5), and nonspecific IVCD in 7 (9.46%) patients. The non‐MD myopathy cohort had no evidence of structural heart disease and showed normal ECG studies (Table 2), making them an appropriate age and gender‐matched (*p* =.08 and *p* =.32, respectively) negative control cohort. Tracked serial ECG data found no statistical difference in the change of parameters among MD patients with cardiomyopathy, MD patients without cardiomyopathy, and patients with non‐MD myopathies (Figure S6).

### 3.4 Assessment of electrocardiogram morphologies

**FIGURE 1:**
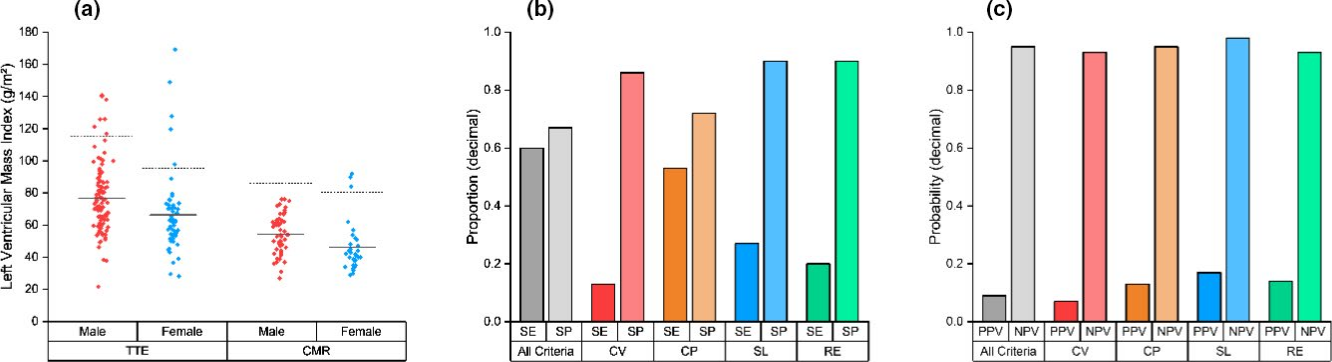
Indexed left ventricular mass of patients by sex and imaging modality with corresponding cutoffs for left ventricular hypertrophy (a), and comparison of conventional criteria for 12‐lead electrocardiogram‐indicated LVH using sensitivity (SE) and specificity (SP) (b), as well as positive predictive value (PPV) and negative predictive value (NPV) (c) in patients with muscular dystrophy. CMR, cardiac magnetic resonance; CP, Cornell voltage‐duration product criteria; CV, Cornell voltage criteria; RE, Romhilt‐Estes point score system; SL, Sokolow–Lyon voltage criteria; TTE, transthoracic echocardiogram

In our study cohort of 203 patients, there were 28 (13.8%) indications of ECG‐LVH by CV (Figure S7 (a)) and 61 (30.0%) indications by CP (Figure S7 (b)), where all indications by CV were found in the presence of CP. Additionally, there were 23 (11.3%) indications by SL (Figure S7 (c)), and 21 (10.3%) indications by RE (Figure S7 (d)). Anatomical LVH was only indicated in 15 (7.39%) patients by cardiac imaging (Figure 1(a)), and therefore, all criteria demonstrated low sensitivity, while demonstrating high specificity (Figure 1(b)). Furthermore, all criteria demonstrated markedly low PPV, while demonstrating high NPV (Figure 1(c)).

**FIGURE 2:**
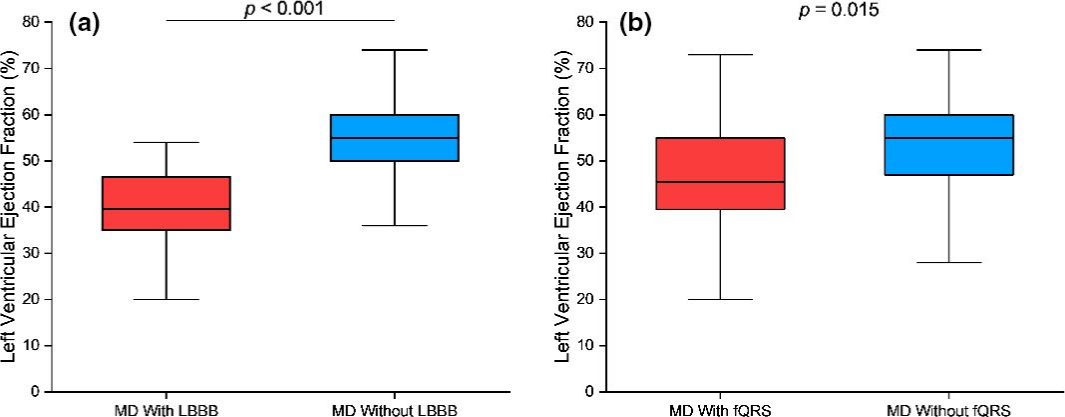
Comparison of left ventricular ejection fraction (LVEF) of muscular dystrophy patients with versus without left bundle branch block (LBBB) (a), and in patients with versus without QRS fragmentation (fQRS) (b)

Left bundle branch block was indicated in the 15 (20.3%) DM1 patients and 2 (5.56%) dystrophinopathies patients. In comparing the Bazett formula versus the Tang and Rabkin formula for QT interval correction, we found intervals of 475.3 (IQR, 438.2–504.1) ms versus 386.0 (IQR, 370.9–430.4) ms (*p *< .001), respectively, in patients with LBBB. Patients with LBBB had a markedly lower LVEF than patients without LBBB (39.6 [IQR, 35.0–46.6] % versus 55.0 [IQR, 50.0–60.0], % respectively; *p *< .001; Figure 2(b)) and were more likely to have a cardiomyopathy (RR, 2.75 [95% CI, 2.14–3.53]; *p *< .001). Patients with LBBB were also more likely to require cardiac medical therapies (RR, 1.86 [95% CI, 1.17–2.96]; *p *= .008) and cardiac device intervention (RR, 12.29 [95% CI, 5.75–26.30]; *p *< .001) than patients without LBBB. There was no discernible difference between patients with or without LBBB and the presence of fibrosis or the incidence of arrhythmias.

QRS fragmentation was indicated in 6 (16.7%) dystrophinopathies patients, 2 (5.56%) LGMD patients, 11 (14.9%) DM1 patients, and 1 (4.35%) FSHD patient. Patients with fQRS had a lower LVEF than patients without fQRS (45.5 [IQR, 39.6–55.0] % versus 55 [IQR, 47.5–60.0] %, respectively; *p *= .015; Figure 2(b)) and were more likely to have a cardiomyopathy (RR, 1.76 [95% CI, 1.20–2.59]; *p *= .004). Patients with fQRS were also more likely to require cardiac medical therapies (RR, 1.74 [95% CI, 1.10–2.77]; *p* = .018) and cardiac device intervention (RR, 4.35 [95% CI, 1.94–9.74]; *p* < .001) than patients without fQRS. There was no discernible difference between patients with or without fQRS and the presence of fibrosis or the incidence of arrhythmias.

**FIGURE 3:**
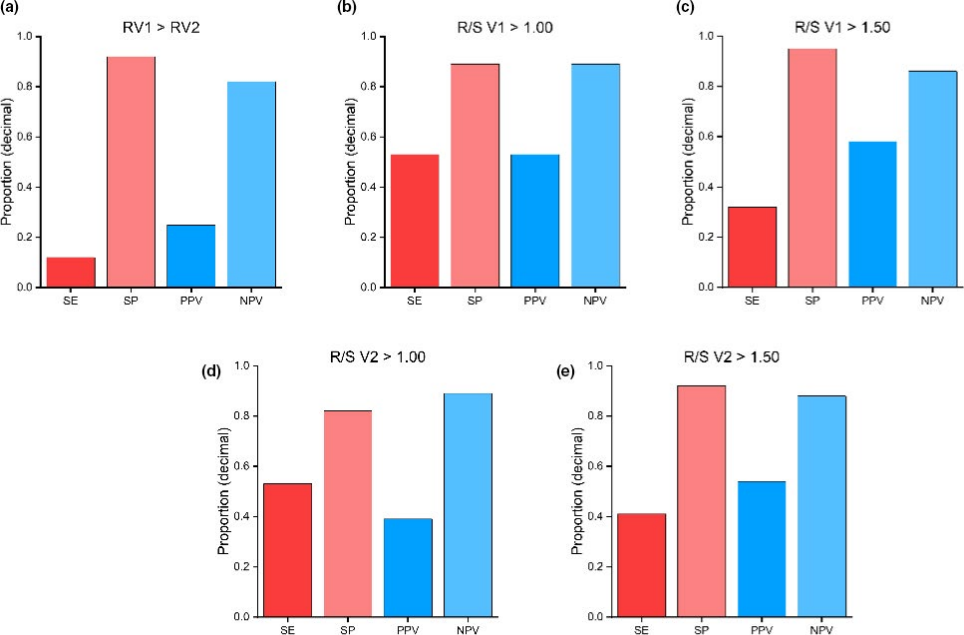
Assessment of variations of R‐wave progression to differentiate dystrophinopathies from other types of muscular dystrophy using sensitivity (SE), specificity (SP), positive predictive value (PPV), and negative predictive value (NPV) of 12‐lead electrocardiogram morphology such as a RV1 greater than RV2 (a), an R/S ratio in V1 greater than 1.00 (b), an R/S ratio in V1 greater than 1.50 (c), an R/S ratio in V2 greater than 1.00 (d), and an R/S ratio in V2 greater than 1.50 (e)

Patterns of R‐wave progression were assessed for their ability to differentiate between patients with or without a dystrophinopathy. Presentation of an R wave of greater amplitude in V1 than V2, an R/S ratio greater than 1.00 and 1.50 in V1, or an R/S ratio greater than 1.00 and 1.50 in V2 demonstrated low sensitivity and high specificity, with low PPV and high NPV (Figure 3(a‐e)).

## 4 DISCUSSION

Heart disease is highly prevalent in patients with MD and is a major determinant of their clinical outcomes. (Nikhanj et al., 2020; Nikhanj, Yogasundaram, et al., 2020) 12‐lead electrocardiogram assessment is easily accessible, requires minimal training, and is of negligible burden on healthcare resources, making it a convenient and feasible method of assessing heart disease. Our investigation evaluated the use of conventional ECG‐LVH criteria in patients with MD. The CV, CP, SL, and RE criteria are conventional methodologies for the identification of LVH through the analysis of voltages and wavelengths, with consideration for gender. We determined that these criteria demonstrated minimal clinical utility in these patients when compared to anatomical measures of LV mass illustrated by markedly low sensitivity and positive predictive value. The high specificity and NPV of the criteria can be useful for the confirmation of LVH indicated by cardiac imaging but would not be independently useful from a diagnostic perspective. Qualification of cardiac hypertrophy in patients with DMD is a greater challenge due to high precordial voltages, namely R waves in V1, though the exact cause remains unknown. (Thrush et al., 2013).

Discordance between ECG‐LVH and anatomical LVH is not uncommon in patients with heart disease and has been well‐documented in large cohort studies. (Kannel et al., 1969; Okwuosa et al., 2015) Our investigation demonstrates a greater degree of disagreement in patients with MD. We noted that the SL criteria, which exclusively considers precordial voltages for the classification of ECG‐LVH, did not have an advantage over the other criteria that consider a composite of limb and precordial voltages among other properties. This supports our clinical findings of global muscle wasting in our cohort, which is an obstacle to the clinical utility of these conventional criteria in patients with MD. Although we were not able to capture the association between cardiac biomarkers and anatomical LVH in this cohort due the low prevalence, we support the proposed prognostic value of comparable B‐type natriuretic peptide and hsTnI for MACE in these patients, independent of LVH. (Nikhanj, Nichols, et al., 2020) We acknowledge that ECG‐LVH could be representative of interstitial and ion channel remodeling in the absence of myocyte hypertrophy. (Aro Aapo & Chugh, 2016) With regard to the indication of LA enlargement by ECG, previous studies have shown that the level of specificity could prove useful for confirmation of indications by imaging but not for diagnosis, similar to our findings with ECG‐LVH. (Ng et al., 2020; Tsao et al., 2008).

Left bundle branch block was an important ECG morphology identified in patients with DMD and DM1 given the high probability of a subsequent diagnosis of cardiomyopathy. Though LBBB was primarily indicated in patients with DM1, patients with DMD were likely also represented due to their advanced progression of heart disease. (Nikhanj, Yogasundaram, et al., 2020) Patients with DM1 were distinguishable given their high prevalence of conduction disease and incidence of arrhythmias, as shown in previous research. (Benhayon et al., 2015; Nikhanj, Miskew‐Nichols, Sivakumaran, et al., 2019; Nikhanj, Sivakumaran, Yogasundaram, et al., 2019) Given that QRS interval progression has been shown to correlate with reduced LV systolic function,^40^ CRT remains an important therapy to restore ventricular synchrony in the setting of LBBB, which can be supported with beta‐blocker therapies in patients with ventricular tachyarrhythmias, as we have previously investigated in patients with DM1. (Nikhanj, Sivakumaran, Yogasundaram, et al., 2019) The severity and progression of conduction disease in patients with DM1 correlate with age and with the quantity CTG repeats, which supports the supplementation of demographics and genetic testing into the risk stratification of patients with DM1 in addition to baseline ECG analysis. (Groh et al., 2002; Nazarian et al., 2011) As we observed, cardiac conduction abnormalities were less prevalent than structural abnormalities in patients with BMD and LGMD. (Petri et al., 2015) Patients with dystrophinopathies did present with uniquely abnormal R‐wave progression, and the various patterns assessed could serve to confirm a diagnosis of a dystrophinopathy given their high specificity. Furthermore, cardiomyopathy secondary to FSHD remains variably reported and any positive findings have included atrial arrhythmias and atrioventricular conduction delays, though electrophysiological assessment of FSHD patients in our study cohort was unremarkable. (Nikhanj, Yogasundaram, et al., 2020).

We note that our study presents a MD cohort with a high prevalence of first‐degree AVB and LAFB consistent with conduction disease in MD patients. (Mathieu et al., 1999) Our clinic has been established and optimized with regional primary care physicians to enroll patients with MD at early stages of their disease course, which is critical to the management of rapidly progressing conduction disease and incidence of arrhythmias. Importantly, VT is common in patients with DMD and DM1 including the risk of sudden cardiac death, which can be mitigated through the use of prophylactic device intervention as provided to our patients. (Nikhanj, Miskew‐Nichols, Sivakumaran, et al., 2019; Nikhanj, Sivakumaran, Yogasundaram, et al., 2019; Nikhanj, Yogasundaram, et al., 2020).

QRS fragmentation has been described as a marker for cardiac fibrosis and a prognostic indicator of MACE in patients with heart disease. (Das Mithilesh et al., 2008; Haukilahti et al., 2016; Terho et al., 2014) Given that fQRS is representative of heterogenous ventricular activation and structural heart disease in traditional cohorts of heart disease, it is reasonable to conclude that MD patients exhibiting this morphology presented with advanced structural heart disease, as reflected in the high probability of cardiac outcomes in our patients with fQRS. (Haukilahti et al., 2016) Considerations of fQRS have previously been applied to patients with DMD as a representation of regional wall motion abnormalities, and as an early indicator of adverse cardiac remodeling in these patients. (Cho et al., 2017; Yoo et al., 2017) We believe that this concept can be applied to our broader MD cohort considering its prevalence and the associated reduction in LV systolic function. Importantly, fQRS has been shown to be a reliable indicator of adverse cardiac remodeling in the presence of confounding ECG findings, which is relevant to our cohort given the high prevalence of conduction abnormalities. (Strauss David et al., 2008) The identification of fQRS in patients with MD upon ECG assessment is therefore important for prompting cardiac imaging, therapeutic intervention, and active monitoring.

### 4.1 **Study** l**imitations**

We acknowledge the limitations of our investigation. Due to our modest cohort size, we were unable to complete a thorough subgroup analysis to compare the prevalence of the aforementioned ECG features and serial parameter changes between the different types of MD. We recognize the importance of serial monitoring of cardiac electrophysiology in patients with DM1 due to the progression of conduction abnormalities and associated reduction in LV function, high incidence of arrhythmias, and risk of sudden cardiac death. (Groh et al., 2002; Nikhanj, Miskew‐Nichols, Sivakumaran, et al., 2019) Taking a rigorous approach to the serial quantification of atypical ECG parameters would be of strong consideration for future studies. Our analysis of ECG morphology provided important insights into cardiomyopathy and the limitations of ECG‐LVH in these patients but did not specifically evaluate electrocardiogram‐indicated right ventricular hypertrophy (ECG‐RVH). Given the high burden of respiratory disease in patients with MD, ECG‐RVH may be indicative of pulmonary hypertension or chronic obstructive pulmonary disease in these patients and these indications would be conducive to the multidisciplinary care of these patients. (Chen et al., 2018; Nikhanj, Yogasundaram, et al., 2020).

## 5 CONCLUSION

Our investigation demonstrates the clinical utility of ECG and the importance of identifying baseline ECG morphologies such as LBBB and fQRS to facilitate active monitoring, further cardiac assessment through imaging modalities, and therapeutic response in patients with various types of MD. We have also demonstrated that ECG‐LVH through the use of conventional criteria is of minimal clinical utility in these patients and that serial monitoring of intervals is not an effective methodology for stratifying MD patients for cardiomyopathy.

## DISCLOSURES

The authors declare that they have no known competing financial interests or personal relationships that could have appeared to influence the work reported in this article.

## CONFLICTS OF INTEREST

The authors report no relevant conflicts of interest.

## Supporting information

Supplementary Material

## Data Availability

The data that support the findings of this study are available from the corresponding author upon reasonable request.
