# Supplementary material for: Clinical utility of 12‐lead electrocardiogram in evaluating heart disease in patients with muscular dystrophy: Assessment of left ventricular hypertrophy, conduction disease, and cardiomyopathy
Source: Ann Noninvasive Electrocardiol. 2021 Jul 11;26(6):e12876. doi: 10.1111/anec.12876 (PMC8588368; doi:10.1111/anec.12876)
Supplement: Supplementary file 1 — Supplementary Material [file ANEC-26-e12876-s001.pdf]

Supplemental Appendix

Nikhanj A, Yogasundaram H, Kimber S, et al. Clinical Utility of 12-Lead Electrocardiogram in Patients with Muscular Dystrophy: Assessment of Conduction Disease and Left Ventricular Hypertrophy.

Figure 1A. Distribution of muscular dystrophy cohort patient diagnoses as confirmed by genetic testing.

Figure 1B. Timeline of cardiac assessment under the neuromuscular multidisciplinary clinic care pathway.

Figure 1C. Quantity of baseline and serial electrocardiograms tracked in the composite patient cohort.

Figure 2. 12-lead electrocardiogram of a 32-year-old male patients with Duchenne muscular dystrophy presenting with sinus rhythm, normal intervals, and QRS fragmentation in the anterior leads.

Figure 3. 12-lead electrocardiogram of a 25-year-old male with Duchenne muscular dystrophy presenting with sinus rhythm, left bundle branch block, and QRS fragmentation in the anterior leads.

Figure 4. 12-lead electrocardiogram of a 46-year-old male with type 1 myotonic dystrophy presenting with sinus rhythm, first-degree atrioventricular block, and QRS fragmentation in the lateral leads.

Figure 5. 12-lead electrocardiogram of a 45-year-old male with type 1 myotonic dystrophy presenting with atrial flutter with 3-to-1 conduction and left bundle branch block.

Figure 6. Comparison of serial 12-lead electrocardiogram parameter changes among muscular dystrophy (MD) patients with cardiomyopathy, MD patients without cardiomyopathy, and patients with non-MD myopathies.

Figure 7A. 12-lead electrocardiogram of an 18-year-old male with Duchenne muscular dystrophy presenting with sinus tachycardia and left ventricular hypertrophy indicated by Cornell voltage and Cornell voltage-duration product criteria.

Figure 7B. 12-lead electrocardiogram of a 79-year-old female with limb-girdle muscular dystrophy presenting with sinus rhythm, a first-degree atrioventricular block and left ventricular hypertrophy indicated by Cornell voltage-duration product criteria.

Figure 7C. 12-lead electrocardiogram of a 30-year-old male with type 1 myotonic dystrophy presenting with sinus rhythm and left ventricular hypertrophy indicated by Sokolow-Lyon criteria.

Figure 7D. 12-lead electrocardiogram of a 24-year-old male with facioscapulohumeral muscular dystrophy presenting with sinus rhythm and left ventricular hypertrophy indicated by Romhilt-Estes point score criteria.

Table 1. Criteria used to define electrocardiogram-indicated left ventricular hypertrophy.

References

Figure 1A. Distribution of muscular dystrophy cohort patient diagnoses as confirmed by genetic testing.

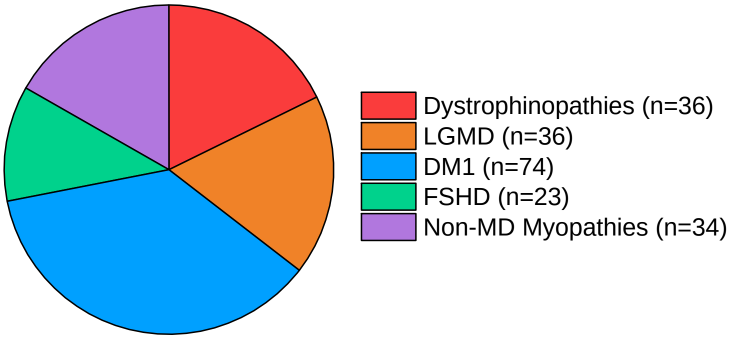

DM1, type 1 myotonic dystrophy; FSHD, facioscapulohumeral muscular dystrophy; LGMD, limb-girdle muscular dystrophy.

Figure 1B. Timeline of cardiac assessment under the neuromuscular multidisciplinary clinic care pathway.

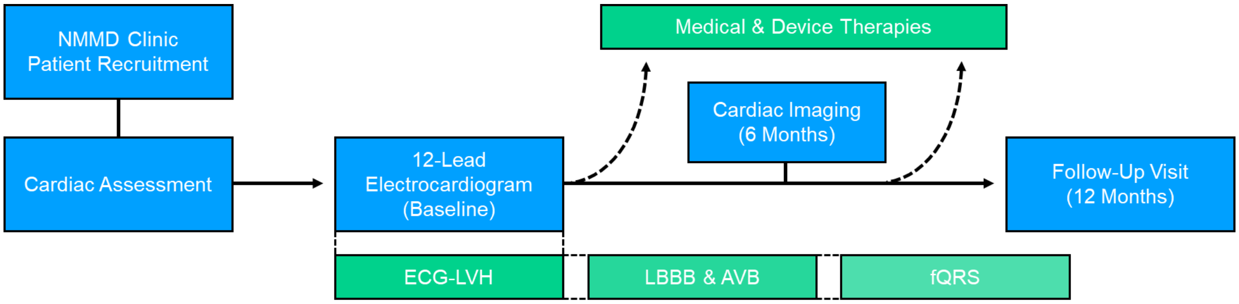

AVB, atrioventricular block; ECG-LVH, electrocardiogram-indicated left ventricular hypertrophy; fQRS, QRS fragmentation; LBBB, left bundle branch block; NMMD, neuromuscular multidisciplinary.

Figure 1C. Quantity of baseline and serial electrocardiograms tracked in the composite patient cohort.

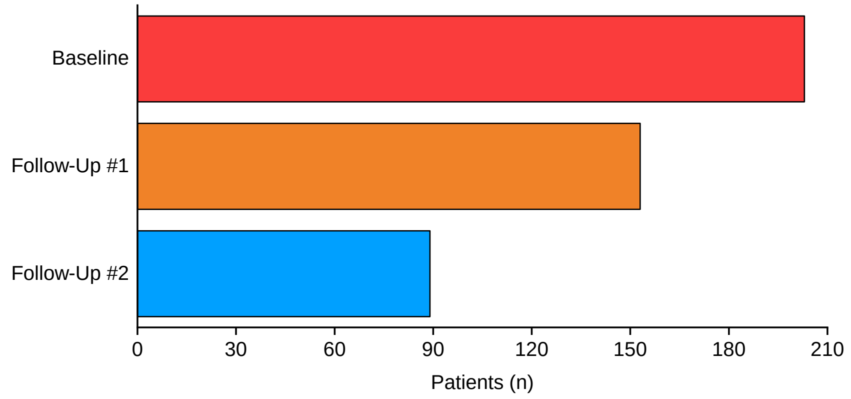

Figure 2. 12-lead electrocardiogram of a 32-year-old male patients with Duchenne muscular dystrophy presenting with sinus rhythm, normal intervals, and QRS fragmentation in the anterior leads.

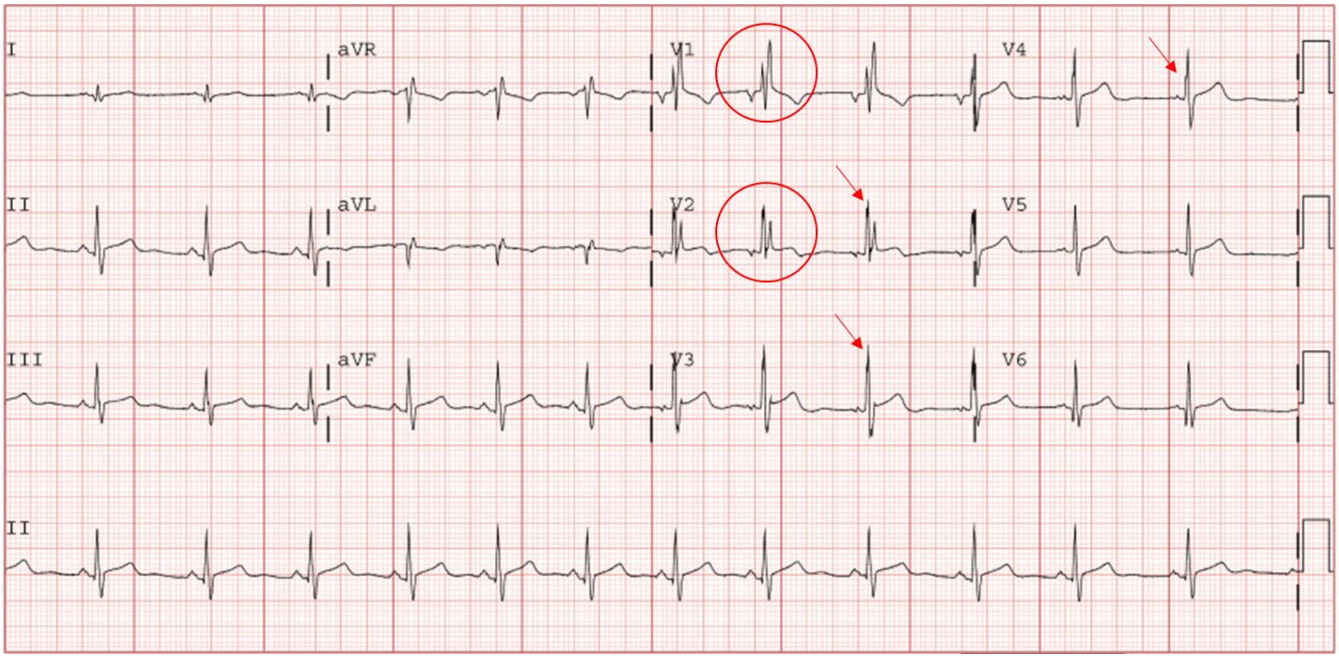

Electrocardiogram indicates a heart rate of 80 bpm, PR interval of 120ms, and QRS duration of 96ms. Circles indicate RSR’ pattern shown in two contiguous anterior leads and arrows indicate notches in the nadir of R waves in three contiguous anterior leads, concordant with our definition of QRS fragmentation.

Figure 3. 12-lead electrocardiogram of a 25-year-old male with Duchenne muscular dystrophy presenting with sinus rhythm, left bundle branch block, and QRS fragmentation in the anterior leads.

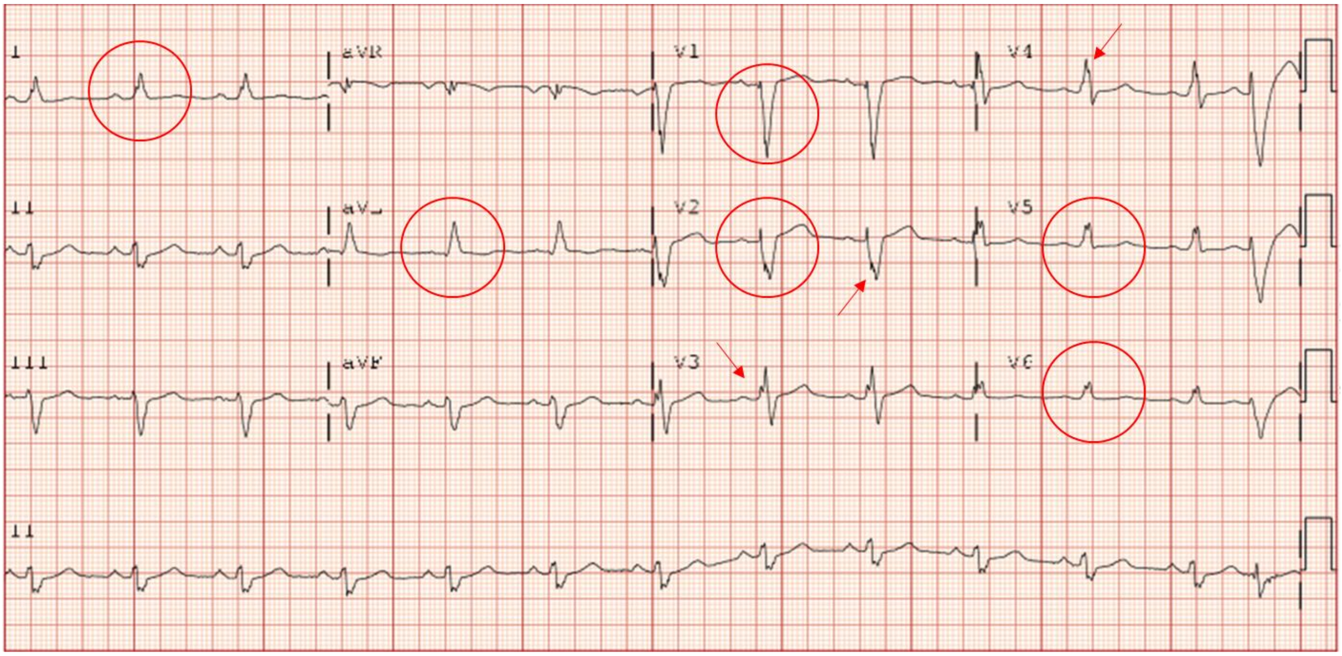

Electrocardiogram indicates a heart rate of 74 bpm, PR interval of 168ms, and QRS duration of 140ms. Circles indicate slurred R waves in leads I and aVL, and RSR’ patterns in V5 and V6 with peak time greater than 60ms, concordant with our definition of left bundle branch block. Arrows indicate notches in the nadir of S and R waves in three contiguous anterior leads, concordant with our definition of QRS fragmentation in the presence of a bundle branch block.

Figure 4. 12-lead electrocardiogram of a 46-year-old male with type 1 myotonic dystrophy presenting with sinus rhythm, first-degree atrioventricular block, and QRS fragmentation in the lateral leads.

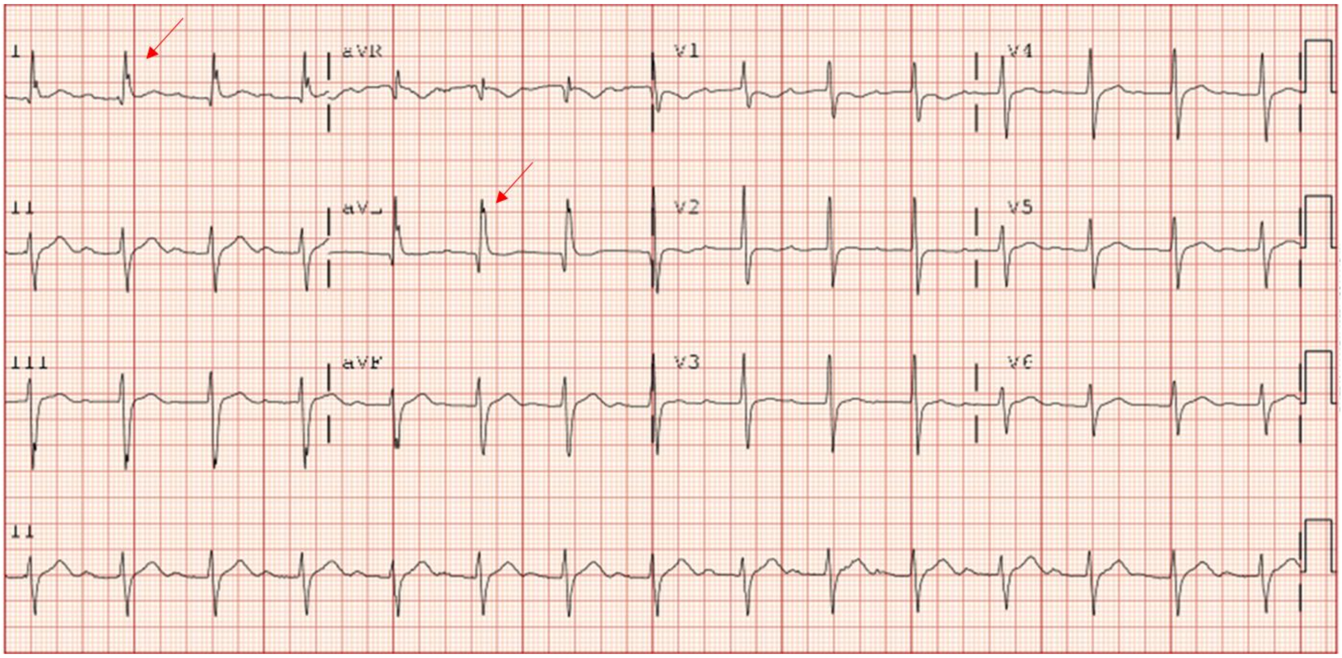

Electrocardiogram indicates a heart rate of 88 bpm, PR interval of 294 ms, and QRS duration of 109 ms. Arrows indicate notches in the nadir of R waves in two contiguous lateral leads, concordant with our definition of QRS fragmentation.

Figure 5. 12-lead electrocardiogram of a 45-year-old male with type 1 myotonic dystrophy presenting with atrial flutter with 3-to-1 conduction and left bundle branch block.

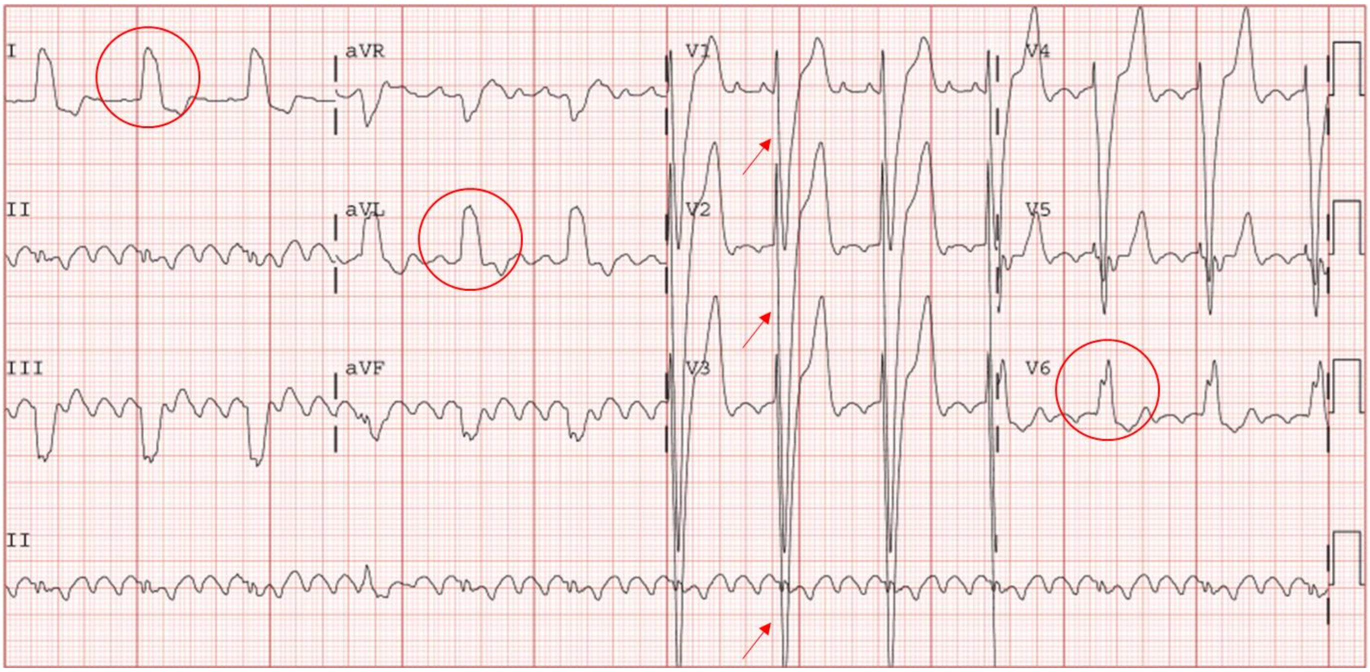

Electrocardiogram indicates a ventricular rate of 75 bpm. Circles indicate slurred R waves in leads I and aVL, and RSR' patterns in V5 and V6 with peak time greater than 60ms, concordant with our definition of left bundle branch block (LBBB), and arrows indicate deep S waves in V1-V3 as a common identifier of LBBB.

Figure 6. Comparison of serial median 12-lead electrocardiogram parameter changes among muscular dystrophy (MD) patients with cardiomyopathy, MD patients without cardiomyopathy, and patients with non-MD myopathies.

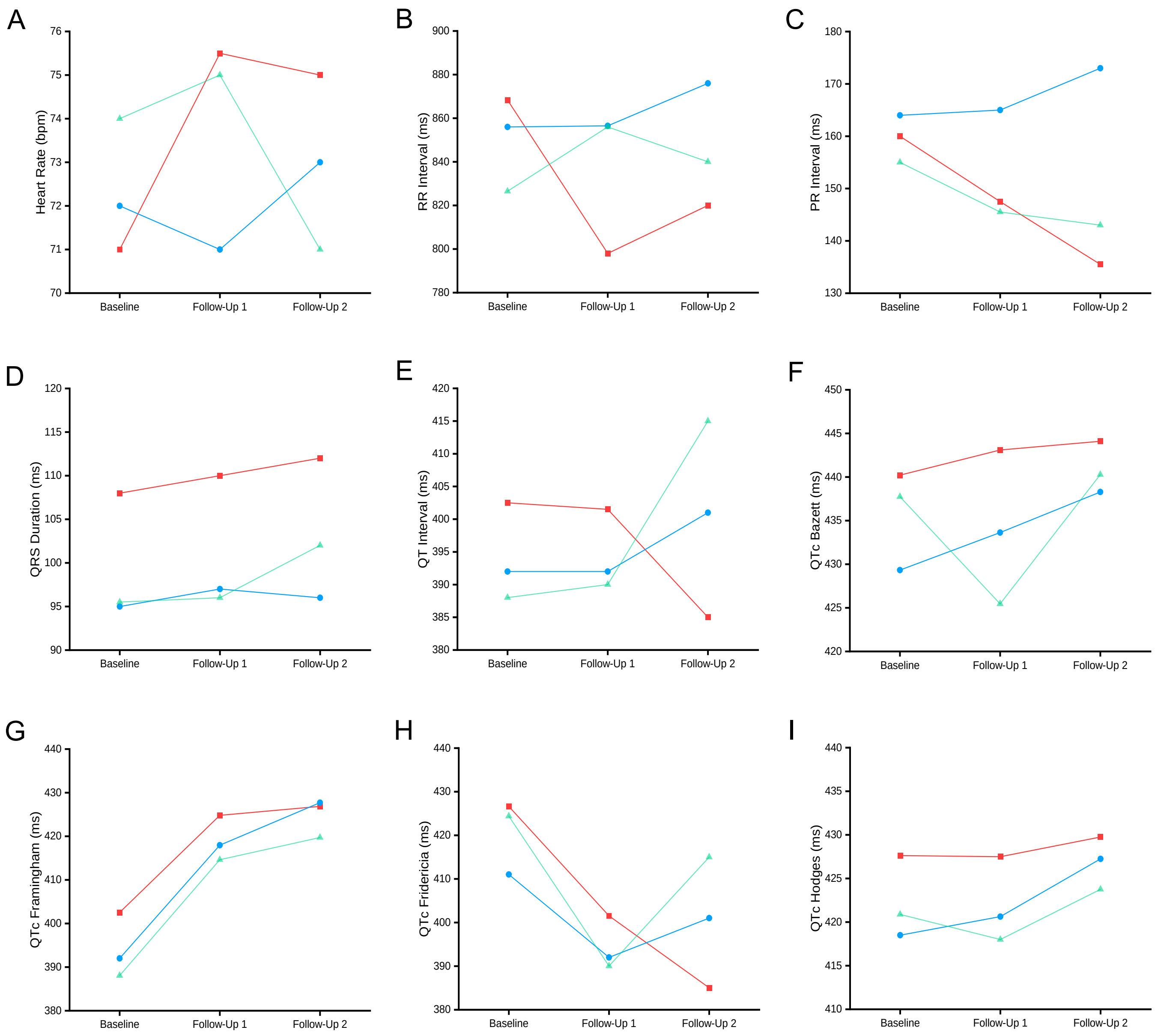

		Baseline	Follow-Up 1	Follow-Up 2
**MD With Cardiomyopathy**	Patients (n)	68	54	37
	Median Follow-Up (Days)	-	382	364
**MD Without Cardiomyopathy**	Patients (n)	101	72	39
	Median Follow-Up (Days)	-	419	366
**Non-MD Myopathies**	Patients (n)	34	27	13
	Median Follow-Up (Days)	-	354	441

Figure 7A. 12-lead electrocardiogram of an 18-year-old male with Duchenne muscular dystrophy presenting with sinus tachycardia and left ventricular hypertrophy indicated by Cornell voltage and Cornell voltage-duration product criteria.

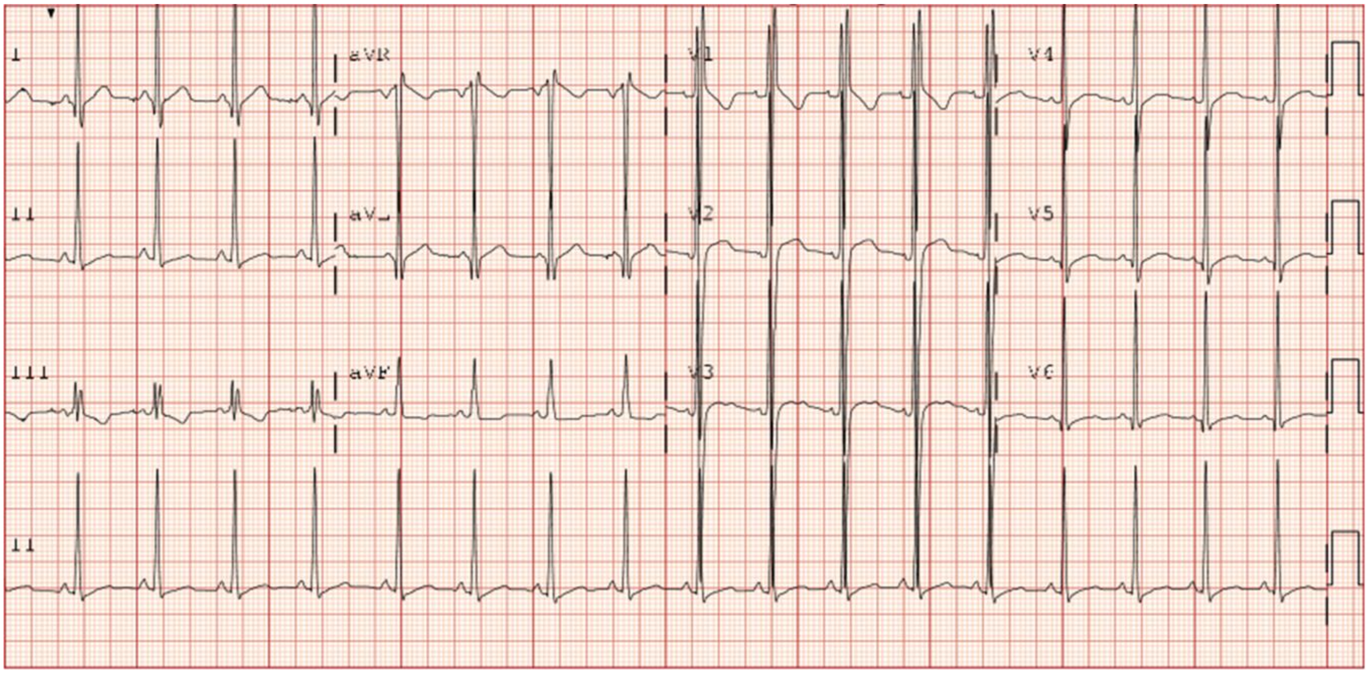

Electrocardiogram indicates a heart rate of 106 bpm, PR interval of 106 ms, and QRS duration of 94 ms. Left ventricular mass index was 63 g/m^2 by transthoracic echocardiogram.

Figure 7B. 12-lead electrocardiogram of a 79-year-old female with limb-girdle muscular dystrophy presenting with sinus rhythm, a first-degree atrioventricular block and left ventricular hypertrophy indicated by Cornell voltage-duration product criteria.

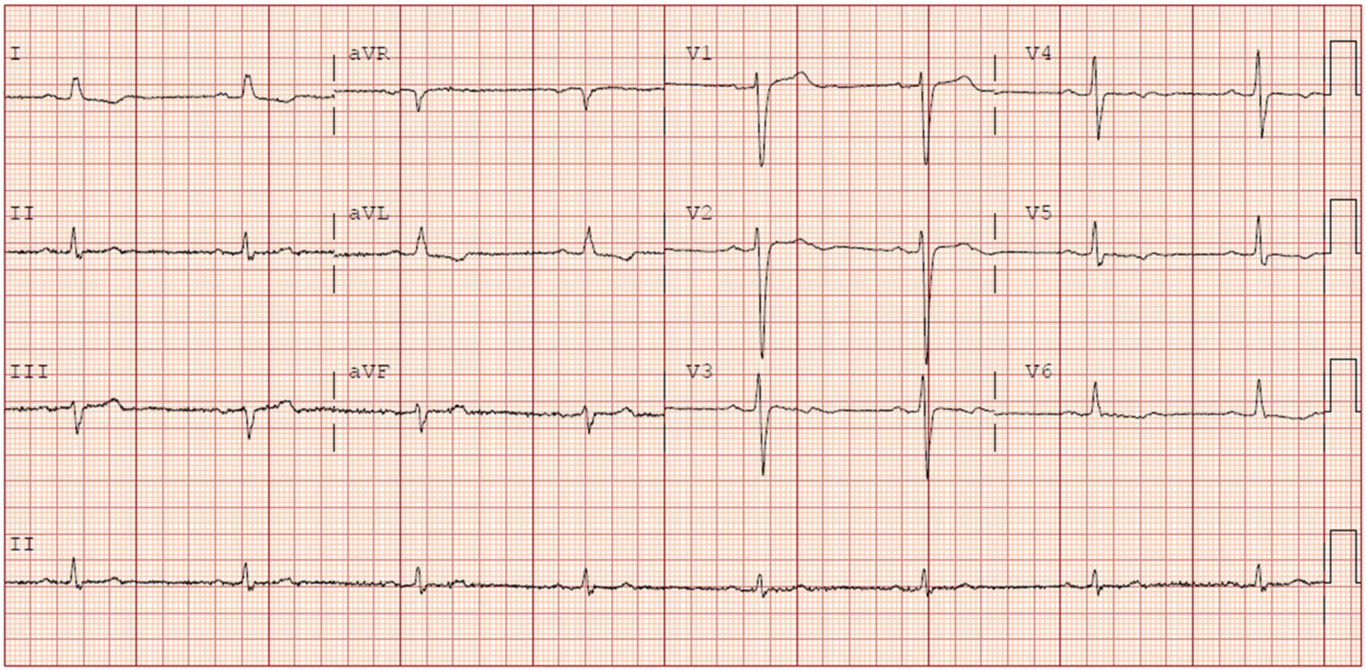

Electrocardiogram indicates a heart rate of 47 bpm, PR interval of 217 ms, and QRS duration of 126 ms. Left ventricular mass index was 48 g/m^2 by cardiac magnetic resonance.

Figure 7C. 12-lead electrocardiogram of a 30-year-old male with type 1 myotonic dystrophy presenting with sinus rhythm and left ventricular hypertrophy indicated by Sokolow-Lyon criteria.

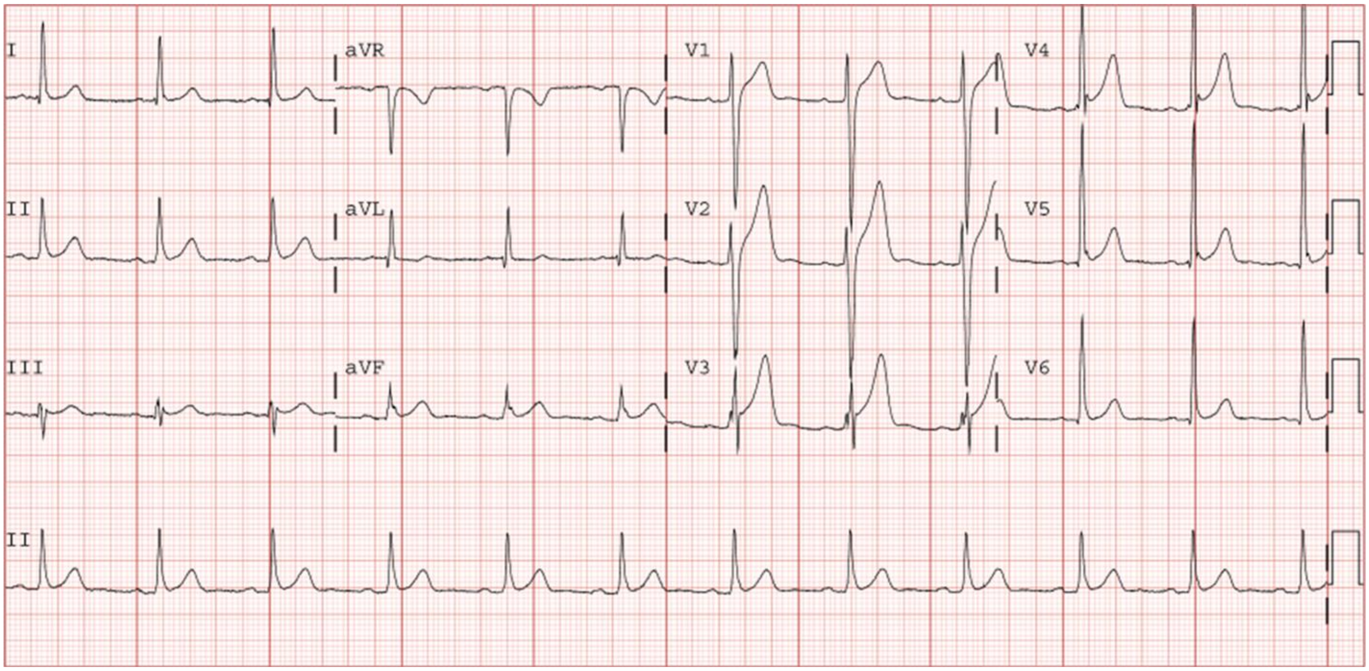

Electrocardiogram indicates a heart rate of 69 bpm, PR interval of 184 ms, and QRS duration of 108 ms. Left ventricular mass index was 62 g/m^2 by cardiac magnetic resonance.

Figure 7D. 12-lead electrocardiogram of a 24-year-old male with facioscapulohumeral muscular dystrophy presenting with sinus rhythm and left ventricular hypertrophy indicated by Romhilt-Estes point score criteria.

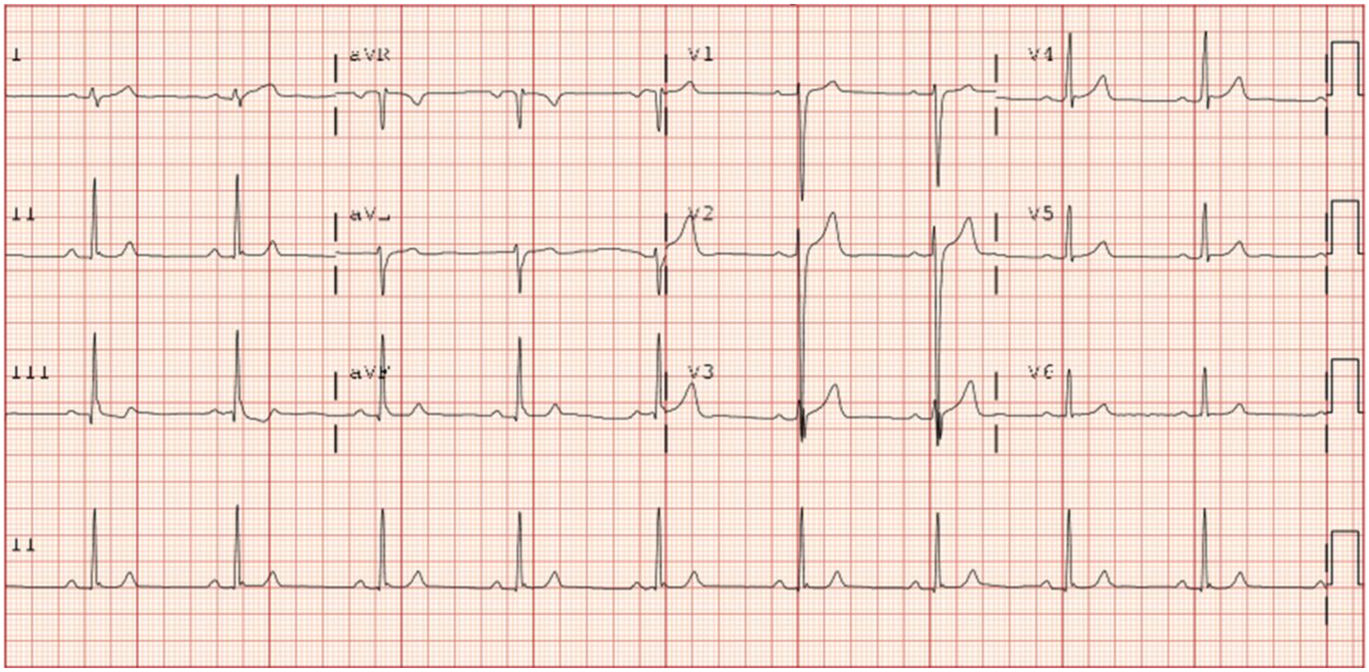

Electrocardiogram indicates a heart rate of 57 bpm, PR interval of 173 ms, and QRS duration of 103 ms. Left ventricular mass index was 44 g/m^2 by cardiac magnetic resonance.

Table 1. Criteria used to define electrocardiogram-indicated left ventricular hypertrophy (LVH).

Criteria	Parameters
Cornell Voltage	RaVL + SV3 > 28 mm (men); > 20 mm (women)[1]
Cornell Voltage-Duration Product	(RaVL + SV3) × QRS duration > 2440 mm x ms (men); (RaVL + SV3 + 80 mm) × QRS duration > 2440 mm x ms (women)[2, 3]
Sokolow-Lyon	SV1 + RV5 or RV6 ≥ 35 mm[4]
Romhilt-Estes Point Score	A. R or S wave in limb leads ≥ 20 mm or SV1 or V2 ≥ 30 mm or RV5 or V6 ≥ 30 mm (3 points), B. ST-T segment pattern without digitalis (3 points) or with digitalis (1 point), C. Left atrial involvement (3 points), D. Left axis deviation more than -30° (2 points), E. QRS duration ≥ 90 ms (1 point), F. Intrinsicoid deflection ≥ 50 sec in V5 or V6 (1 point); A + B + C + D + E + F > 4 points[5]

References

1. Casale PN, Devereux RB, Kligfield P, Eisenberg RR, Miller DH, Chaudhary BS and Phillips MC. Electrocardiographic detection of left ventricular hypertrophy: Development and prospective validation of improved criteria. J Am Coll Cardiol. 1985;6:572-580.
2. Okin PM, Roman MJ, Devereux RB and Kligfield P. Electrocardiographic identification of increased left ventricular mass by simple voltage-duration products. J Am Coll Cardiol. 1995;25:417-423.
3. Molloy TJ, Okin PM, Devereux RB and Kligfield P. Electrocardiographic detection of left ventricular hypertrophy by the simple QRS voltage-duration product. J Am Coll Cardiol. 1992;20:1180-1186.
4. Sokolow M and Lyon TP. The ventricular complex in left ventricular hypertrophy as obtained by unipolar precordial and limb leads. Am Heart J. 1949;37:161-186.
5. Romhilt DW and Estes EH, Jr. A point-score system for the ECG diagnosis of left ventricular hypertrophy. Am Heart J. 1968;75:752-758.
